# Malaria transmission and insecticide resistance of *Anopheles gambiae *in Libreville and Port-Gentil, Gabon

**DOI:** 10.1186/1475-2875-9-321

**Published:** 2010-11-11

**Authors:** Jean-Romain Mourou, Thierry Coffinet, Fanny Jarjaval, Bruno Pradines, Rémi Amalvict, Christophe Rogier, Maryvonne Kombila, Frédéric Pagès

**Affiliations:** 1 Département de Parasitologie-mycologie, Faculté de médecine, Université des Sciences de la Santé, B.P. 4009 Libreville Gabon; 2 UMR 6236, Unité d'entomologie médicale, IRBA antenne Marseille, Allée du Médecin Colonel Jamot, Parc du Pharo, BP 60109, 13262 Marseille Cedex 7, France; 3 UMR 6236, Unité de parasitologie, IRBA antenne Marseille, Allée du Médecin colonel Jamot, parc du Pharo, BP 60109, 13262 Marseille Cedex 7, France

## Abstract

**Background:**

Urban malaria is a major health priority for civilian and militaries populations. A preliminary entomologic study has been conducted in 2006-2007, in the French military camps of the two mains towns of Gabon: Libreville and Port-Gentil. The aim was to assess the malaria transmission risk for troops.

**Methods:**

Mosquitoes sampled by human landing collection were identified morphologically and by molecular methods. The *Plasmodium falciparum *circumsporozoïte (CSP) indexes were measured by ELISA, and the entomological inoculation rates (EIR) were calculated for both areas. Molecular assessments of pyrethroid knock down *(kdr*) resistance and of insensitive acetylcholinesterase resistance were conducted.

**Results:**

In Libreville, *Anopheles gambiae s.s*. S form was the only specie of the *An. gambiae *complex present and was responsible of 9.4 bites per person per night. The circumsporozoïte index was 0.15% and the entomological inoculation rate estimated to be 1.23 infective bites during the four months period. In Port-Gentil, *Anopheles melas *(75.5% of catches) and *An. gambiae s.s*. S form (24.5%) were responsible of 58.7 bites per person per night. The CSP indexes were of 1.67% for *An. gambiae s.s *and 0.28% for *An. melas *and the EIRs were respectively of 1.8 infective bites per week and of 0.8 infective bites per week. Both *kdr-w *and *kdr-e *mutations in *An. gambiae *S form were found in Libreville and in Port-Gentil. Insensitive acetylcholinesterase has been detected for the first time in Gabon in Libreville.

**Conclusion:**

Malaria transmission exists in both town, but with high difference in the level of risk. The co-occurrence of molecular resistances to the main families of insecticide has implications for the effectiveness of the current vector control programmes that are based on pyrethroid-impregnated bed nets.

## Background

Malaria remains a major endemic disease in tropical areas and a significant health threat to travellers and military personnel [1]. Since 2003, French forces have implemented many studies to assess the malaria risk according to the locations of troops and the insecticide susceptibility of vectors in the field [2-4]. In Gabon, malaria is predominantly caused by *P. falciparum *and malaria transmission is perennial and was considered until today as hyperendemic [5]. Throughout the country, infections with *P. falciparum *accounted for 30% to 50% of the consultations of children with fever between 1980s and 1990s [6,7]. In Libreville during the same period, the prevalence of *P.falciparum *infection and of anemia were respectively of 57% and 71% in pregnant women [8] In 2005, artemisinin-based combination therapy (ACT), insecticide- treated nets (ITNs) and intermittent preventive treatment (IPT) were freely available for children under five years old and pregnant women [9]. Since then, a decline of malaria burden has been described in Libreville both in pregnant women and children [9,10]. Actually, parasitaemic data in febrile children over the age of five years are questioning about both the impact on the level of transmission in Libreville and on the acquisition of malaria immunity in urban areas by children [11]. Libreville is probably an urban area in which a shanty-town with high malaria transmission and a residential area with low transmission coexist. Unfortunately, none recent entomologic data exist on malaria transmission in Libreville or other major towns of the country. Studies conducted in the inlands showed that malaria transmission was sustained mainly by members of the *Anopheles gambiae species complex *and the *Anopheles funestus Giles species complex *[5,12,13]. In Libreville, a study conducted during the rainy season in 2000 showed the presence of *An. gambiae s.s. molecular form S *and enhanced the presence of both *kdr-e *and *kdr-w *alleles at high frequencies in this population [14].

Malaria is a major health priority for French forces in Gabon since many years and always today. Many cases have occurred and many outbreaks have been described in the country or in returnees [15,16]. So most of the last clinical trials on alternative chemoprophylaxis for troops have been conducted in Gabon [17,18]. The vector control programs in the camps include personal protection measures (PPMs) and unit protection measures (UPMs). PPMs consist of proper use of uniforms, the application of repellent to exposed skin, the application of permethrin to battledress uniforms, and the use of insecticide-impregnated bed-nets. UPMs include environmental management, larviciding, and space spraying. Here are reported the results of a preliminary entomologic study conducted in Equatorial Africa in the two French military camp in Gabon: "camp de Gaulle" in Libreville, the capital city, and "camp N'tchorere" in Port-Gentil the economic capital city. The aim of this study was to assess the local malaria transmission risk for troops in these urban areas.

## Methods

### Location

This prospective and observational study was conducted from December 2006 to April 2007 in Libreville (0°23'24"N 9°26'59"E) and in February 2007 in Port-Gentil (-0°43'11''N 8°46'47''E). Libreville, the capital city of Gabon, is situated on the borders of the guinea gulf in West central Africa and on the border of the Komo River. The population of Libreville was estimated to be 537,540 inhabitants in 2006. The "camp de Gaulle" is situated at the north of town near the international airport (0°26'32''N, 9°25'38''E). It is surrounded by a forested swamp on its north, with habitations including middle class and poor populations and garden markets on the other sides. Port-Gentil is the second largest city of Gabon and is situated on the Mandji Island in the delta of the Ogoué River. The population of Port-Gentil is estimated to 120 600 inhabitants. The "camp N'tchorere" (-0°42'57''N, 8°45'37'') is situated in a periurban area and is surrounded on its west and south sides by forested or deforested areas that are liable to flooding during the rainy season and on the others sides by a few number of modern and traditional habitations.

### Climate

Libreville and Port-Gentil features a tropical monsoon climate with a lengthy wet season (September through May) and a short dry season (June through August). Average temperatures remain relatively constant throughout the course of the year, with average high temperatures at around 30°C.

### Field mosquito processing

Sampling by human landing of malaria vectors was carried out both indoors and outdoors. Collectors gave prior informed consent and received anti-malaria prophylaxis and yellow fever immunization. During this survey, it was very difficult according to military constraints and the organization of the camps (modern habitat totally closed) to find indoor catching point that was representative of the indoor malaria transmission risk and acceptable for military authorities. That's why most of mosquito collections have been done outdoor. In Libreville, landing catches were performed at six points (one place indoors and five outdoors) from January 2007 to March 2007 during 23 nights and at another outdoor point during 8 nights (i.e. 146 person-nights). In Port-Gentil, human landing collections were performed in February 2007 during three nights (i.e. eight person-nights): at four points the first night (one indoor and three outdoor locations) and at two outdoor locations during the two last nights. They were sorted by genera, and anopheline and Culicinae mosquitoes were identified morphologically [19,20]. All mosquitoes were stored individually in numbered vials with desiccant and preserved at -20°C until processing at the Medical Entomology Unit of the Institute for Biomedical research of the French Forces (IRBA), Marseille (France).

### Laboratory mosquito processing

Heads and thoraces of anopheline females were tested by enzyme-linked immunosorbent assay (ELISA) for *P. falciparum *circumsporozoite protein (CSP) [21]. In each site, a random sample of females belonging to the *Anopheles gambiae *complex, together with all CSP-positive anopheline, were identified by polymerase chain reaction (PCR) at the species and molecular forms levels [22]. Molecular characterizations of the *kdr *and *ace1 *mutations were carried out on these mosquitoes as previously described [23,24].

### Data analysis

The human biting rate (HBR) was expressed as the number of female anopheline bites per human per night. The CSP index was calculated as the proportion of mosquitoes found to be positive for CSP. The entomological inoculation rate (EIR) was calculated as the product of the HBR and the CSP index of mosquitoes collected on humans. Confidence interval were calculated by the binomial exact method.

## Results

### Libreville

**Figure 1:**
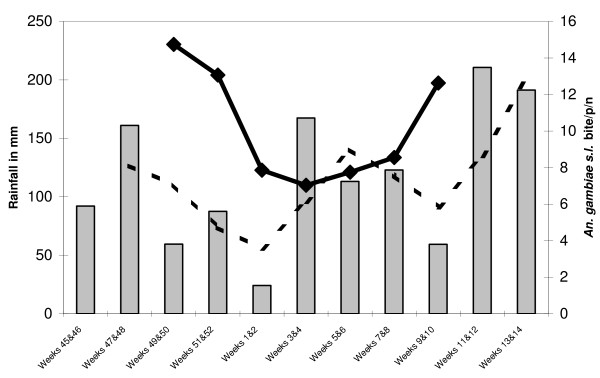
**Evolution of rainfall and of *An. gambiae s.l*. biting rate by periods of two weeks in Libeville, Gabon, from the 45th week of 2006 to the 14th week of 2007**. The gray bars represent the rainfall, the black line represents the number of *An.gambiae s.l*. bites per person per night and the dotted line represents the moving average graph of rainfalls.

**Figure 2:**
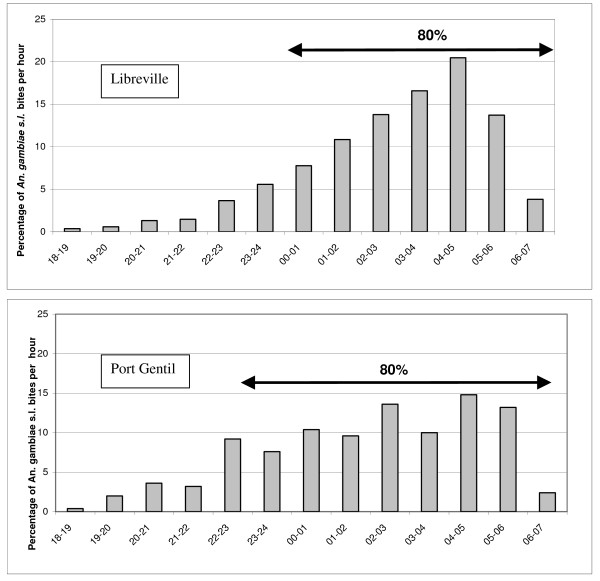
**Distribution by hours of *An. gambiae s.l. bites *in the French military camps of Libreville and Port-Gentil, Gabon 2007**. Gray bars represent the percentage of bites by hour of *An. gambiae s.l.*

In total, 29,076 mosquitoes were caught (94.5% *Culex quinquefasciatus*, 4.7% *Anopheles gambiae s.l.*, 0.3% *Mansonia spp.*, 0.2% *Culex spp.*, 0.2% *Aedes aegypti *and 0.1% *Aedes albopictus*) (Table 1). Overall, the human landing catches gave an average biting rate of 9.3 *An. gambiae s.l*. bites per person per night. The biting rate changed all along the study according to the variations in rainfall: a decrease of rainfall during a two week period was followed by a decrease in *An. gambiae s.l*. biting rate in the following two weeks (Figure 1). Reported to the number of catching points indoor and outdoor, 54.8% of *An. gambiae s.l*. biting rate occurred indoor, indicating that this species was equally endophagic as exophagic. The distribution of *An. gambiae s.l*. bites by hours is shown in Figure 2. The peak biting time both outdoors and indoors was between 24:00 and 6:00 a.m.: 80% of bites occurred during this period. In total, 1,368 *An. gambiae s.l*. collected by human landing catch were processed by ELISA for *P. falciparum *antigen detection. Two specimens were positive: one indoor and one outdoor. The CSP index was 0.15% (CI95% [0.02-0.53]). The weekly EIR was calculated as 0.1 infective bites per week for a person who did not protect himself corresponding to a DOD EIR of 1.23 infective bites per time of deployment on overseas (DOD). On the 375 specimens randomly selected and the two CSP-positive specimens, all were *An. gambiae s.s. molecular form S*. On the 250 specimens analysed for the presence of *kdr-w *and *kdr-e *alleles and *ace1 *mutation, two failed to amplify for the kdr allele. The genotypic frequencies are shown in Table 2. *Kdr-e *and *kdr-w *resistance alleles were present in S forms with a higher frequency of the *kdr-w *allele (59.4%) than the *kdr-e *allele (40.3%). The *ace1 *mutation frequency was 0.4.

**Table 1:** Mosquitoes collected in the French military camps of Gabon in 2007: distribution by species and places of capture (indoor or outdoor)

Species	"Camp de gaulle" Libreville						"Camp N'Tchorere" Port-Gentil					
		
	Indoors		Outdoors		Total		Indoors		Outdoors		Total	
	23 person-nights		123 person-nights		146 person-nights		1 person-nights		7 person-nights		8 person-nights	
						
	*n*	*HBR*	*n*	*HBR*	*n*	*HBR*	*n*	*HBR*	*n*	*HBR*	*n*	*HBR*
*An. gambiae s.l.*	255	11,1	1113	9,0	1368	9,3	19	19	451	64,4	470	58,7
*Culex quinquefasciatus*	3943	171,4	23542	191,4	27485	188,3	17	17	1528	218,3	1545	193,1
*Culex spp.*	2	0,1	60	0,5	62	0,4	11	11	1724	246,3	1735	216,9
*Mansonia spp.*	9	0,4	64	0,5	73	0,5	48	48	2592	370,3	2640	330
*Ae. aegypti*	6	0,3	55	0,4	61	0,4	1	1	5	0,7	6	0,7
*Ae. albopictus*	2	0,1	25	0,2	27	0,2	0	0	0	0	0	0

Total	4217	183,3	24859	202,1	29076	199,2	96	96	6300	900	6396	799,5

**Table 2:** Kdr and AChE genotype frequencies in An. gambiae s.s. molecular form S in Libreville and Port-Gentil, 2007

	n	Kdr genotypes						P***	AChE genotypes			P****
		
		S/S	S/Rw	S/Re	Rw/Rw	Re/Re	Rw/Re		S/S	S/R	RR	
Libreville	250*	0	1	0	80	33	134		248	2	0	
Port gentil	50**	0	1	0	21	3	25	NS	50	0	0	NS

* Two *An. gambiae s.s *consistently failed to amplify for the kdr allele.

** Four *An. gambiae s.s *consistently failed to amplify for the kdr allele.

*** Khi^2^ test

****Fisher test

### Port-Gentil

In total, 6,396 mosquitoes were caught (41.3% *Mansonia spp.*, 27.1% *Culex spp*., 24.2% *Cx quinquefasciatu*s, 7. 3% *An. gambiae s.l*. and 0.1% *Ae. aegypti*) (Table 1). Overall, the human landing catches gave an average biting rate of 58.7 *An. gambiae s.l*. bites per person per night. Reported to the number of catching points indoor and outdoor, 20% of *An. gambiae s.l*. biting rate occurred indoor, indicating that this species was more exophagic. The distribution of *An. gambiae s.l*. bites by hours is shown in Figure 2. The peak biting time indoors was between 22:00 and 6:00 a.m. In total, three specimens on the 470 *An. gambiae s.l*. processed by ELISA were positive for *P. falciparum*: CSP index of 0.63% (CI95% [0.13-1.85]). The weekly EIR was calculated as 2.6 infective bites per week for a person who did not protect himself during the week of study. 209 specimens randomly selected and the three CSP-positive specimens were identified to species level by rDNA-PCR. *Anopheles gambiae s.s*. molecular form S. and *Anopheles melas *were the only two members of the *An. gambiae *complex to be identified. The S-molecular form of *An. gambiae s.s*. accounted for 25.5% of molecular identifications (n = 54) and *An. melas *accounted for 74.5% (n = 158). Within the three CSP-positive specimens, two were *An. gambiae s.s*. molecular form S and one *An. melas*. The circumsporozoïte indexes were respectively of 1.67% (CI95% [0.20-5.89]) for *An. gambiae s.s *and 0.28% (CI95% [0.03-1.02]) for *An.melas*. The respective weekly EIR for a person who did not protect himself during the week of study were of 1.8 infective bites per week for *An. gambiae s.s *and of 0.8 infective bites per week for *An. melas*. We tested all the 54 PCR-identified *An. gambiae s.s *and 96 of the PCR-identified *An.melas *for the *kdr *and *ace1 *mutations. The genotypic frequencies are shown in Table 2. *Kdr-e *and *kdr-w *resistance alleles were present in S forms with a higher frequency of the *kdr-w *allele (68%) than the *kdr-e *allele (31%). None *Kdr *mutation was found in *An.melas*. Both *An.gambiae s.s*. and *An.melas *tested for insensitive AChE were found to be susceptible.

## Discussion

Mosquito population densities were remarkable in the two camps both indoor and outdoor. This is particularly true in Port Gentil regarding the number of *Culex *and *Mansonia *species caught outdoors and the *An.gambiae s.l*. HBR of 58.7 bites per person per night. In Libreville, most of the mosquito biting rate was due to *C.quinquefasciatus*, a pest that breeds easily in waste water. Catches were particularly high near sanitary toilets suggesting that breeding sites were close in the septic tanks. This nuisance could probably be controlled with little effort in improving the airtightness of septic tanks. *Aedes *population densities were low in our study. Although night captures are not appropriate for such diurnal species, these results are concordant with a specific study conducted with BG-sentinel traps to monitor the *Aedes albopictus *and *Ae. aegypti *population in the Libreville camp [25]. *Anopheles gambiae s.l*. HBRs were low ranging from 2.3 to 16.8 bites per person per night during the study period. Figure 1 show the temporal variations in adult density and rainfall during the study. The curve of *An. gambiae s.l*. HBR follow the variations of the moving average graph of the rainfall regime indicating that *An. gambiae *density in Libreville is linked to the intensity of rainfall and probably to the number of temporary breeding sites. The assessment of the endophilic or exophilic behavior could have been biased by the scarcity of indoors collections and the difference between the camps have to be confirmed.

In Libreville, only one species of the *An. gambiae complex *was present: *An. gambiae s.s*. molecular form *S *as already described [14]. The exclusive presence of S form is consistent with the importance of rain-dependent temporary breeding sites in Libreville [26]. In Port Gentil, *An. gambiae *complex was represented by *An. melas *that accounted for two thirds of catches and *An. gambiae s.s*. molecular form S for the last third.

In Libreville, *An. gambiae s.s*. molecular form S is the main malaria vector. According to the *An. gambiae s.s*. density-rainfall association malaria transmission is expected to be lower during the dry season from June to August. Considering the results of this survey as representative of the transmission during rainy season, an estimation of a minimal mean annual EIR of 3.7 infected bite per person per year is realistic. The present observations are consistent with the results of a meta-analysis of studies of malaria transmission in sub-Saharan Africa, which found a mean annual EIR of 7.1 in the city centres, with more than two thirds of the studies reporting EIRs < 4/year [27]. In Port-Gentil, *An. gambiae s.s*. molecular form S and *An. melas *were both involved in *P. falciparum *transmission. If *An. gambiae s.s*. molecular form S accounted for only one third of *An. gambiae s.l*. bites, it was responsible of two third of the *P. falciparum *transmission. Despite its lower vector competence for *P. falciparum, An. melas *contribution in malaria transmission in the Port-Gentil area was high as already described [28,29]. Extrapolating the present findings to the nine months of the rainy season and considering a low transmission level during the dry season, the average annual EIR would be at least of 100 infected bites per person per year. The level of transmission in peri-urban Port-Gentil is similar at those encountered in rural settlements [27]. The malaria transmission is totally different between the two French camps in Gabon. During a four months duty, each platoon of soldiers was based during two weeks in the N'tchorere camp and was clearly exposed to a high number of *P. falciparum *infected bites. According to the delay of incubation, a soldier that doesn't take his chemoprophylaxis would have his malaria attack in Libreville. This situation could have lead to an overestimation of malaria burden for troops in Libreville. In other hand, few cases of malaria occurred in soldiers that were permanently stationed in Port-Gentil for two years. Perhaps the high level of nuisance, nearly 800 mosquito bites per night in February 2007, enhanced the use of personal protection measures by soldiers (i.e. repellents use, bed nets use and proper wearing of battle dress) and their compliance to malaria chemoprophylaxis. 80% of bites are occurring in the second part of the night. During this period, most of soldiers are sleeping in mosquito-proofs buildings and are so protected. Only the outside sentinels are probably exposed to mosquito bites and to malaria vectors.

As reported previously in Gabon in Libreville and in the inlands, both kdr-w and kdr-e mutations in *An. gambiae *molecular form S were present [14; 30]. Contrarily to a previous study in Libreville, *kdr *allele was present both in Libreville and Port-Gentil, and as already described in neighbouring countries (Equatorial Guinea and Cameroon), the kdr-w allele was the most frequent both in Libreville and Port-Gentil [31,32]. The distribution of the *kdr *alleles was similar in the two military camps of Port-Gentil and Libreville but there was a statistically significant difference with the historical data in Libreville (Chi^2^ with 2 df, P < 10^7^). This difference is possibly due to sampling bias or to variations in the repartition of *kdr *alleles in *An. gambiae s.s*. populations between quarters in Libreville. Both *kdr-w *and *kdr-e *mutations have been tightly linked with DDT and pyrethroid resistance phenotypes in field populations of *An. gambiae s.s *[33-36]. The presence of both *kdr-e *and *kdr-w *alleles at high frequencies in these populations has implications for the effectiveness of the current vector control programmes, that are based on pyrethroid-impregnated bed nets both for French forces and for the Libreville or Port-Gentil inhabitants. Currently, the impact of *kdr *on the efficacy of ITNs is unclear with contradictories studies [37-39]. Nevertheless kdr screening is still the best molecular diagnostic tool for predicting pyrethroid and DDT efficacy in preliminary studies like this one [40]. Therefore, before interventional studies, a standard WHO exposure to DDT or permethrin has to be conducted to clearly assess the resistance level and take into account the possible involvement of additional resistance mechanisms.

It was the first time that a molecular screening was conducted in Gabon to assess the presence of an insensitive acetylcholinesterase (AchE) in populations of *An. gambiae*. A low level of insensitive AChE was detected in Libreville with an allele frequency of 0.4. None insensitive AChE was detected in Port-Gentil but there was no statistically difference in the ace1 mutation frequency between Libreville and Port-Gentil. Complementary studies have to be conducted to monitor the possible existence of a resistance to organophosphates and carbamate insecticides in urban *An.gambiae s.s*. form S populations in Gabon. If carbamates remain a viable alternative in front of the low level of insensitive AchE and in the Gabonese context of a probable high resistance to pyrethroids, their extensive use alone would probably conduct to select multi-resistant specimens of *An.gambiae s.s*. form S in Libreville and Port-Gentil. This possibility has to be taking in account before implementing vector control programmes both in military camps and in towns. The use of a combination of pyrethroids and organophosphates in mixture, mosaic or in parallel on different supports has been proposed against pyrethroid-resistant malaria vectors [41-43]. A new mosaic long-lasting insecticidal net developed to control wild pyrethroid resistant *An. gambiae has *shown no benefits compared to classical long-lasting insecticidal nets [44,45].

According to literature, this was the first time that entomological studies on malaria transmission were conducted in the main towns of Gabon, which represent more than 50% of the total population. Malaria transmissions exist in both towns but with high difference in the level of risk. In urban areas, malaria transmission is highly focused and a single study in one area of a town is not sufficient to assess the global level of transmission. And one should not minimize the possible impact of the vector control programme implemented in the camps all year round. In Libreville, the risk is comparable to other big African cities, but seems very low according to the equatorial situation and the previously described hyperendemic transmission. Nevertheless, these results are concordant with the clinical statements made by physicians and researchers in Libreville that all concluded to a decrease in malaria transmission since 2000 in Libreville [9-11]. In Port-Gentil, the level of transmission is high but is probably not representative of the current situation in all parts of Port-Gentil. *Anopheles melas *is clearly involved in malaria transmission in Port-Gentil, but further studies in different parts of Libreville are necessary to understand its involvement or not. Until today, molecular screenings of resistance in Gabon have only concerned kdr mutation. Here is the first report of insensitive acetylcholinesterase in Gabon. This paper highlights the worrying level of pyrethroids resistance of *An. gambiae s.s*. for most of 50% of Gabon's population. There is a lack of entomological data to understand the dynamic of urban malaria in Gabon. Further studies have to been conducted in different areas of theses towns including central and peripheral districts, coastal and inland districts, modern and traditional districts. The situation of insecticide resistance has to be clarified in using molecular tools and insecticide bioassays in different districts of each town.

## Conflict of interest

The authors declare that they have no competing interests.

## Authors' contributions

JRM was responsible for the supervision of data collection, analysis, interpretation and production of the final manuscript and revisions. TC contributed to the supervision of data collection, the data analysis, interpretation and production of final manuscript. FJ contributed to the supervision of data collection, to the data analysis. BP contributed to the supervision of data collection, to the data analysis, interpretation and production of final manuscript. RA FJ contributed to the supervision of data collection, to the data analysis. CR contributed to overall scientific management, analysis, interpretation and preparation of the final manuscript and revisions. MK contributed to overall scientific management, analysis, interpretation and preparation of the final manuscript and revisions. FP was responsible for overall scientific management, analysis, interpretation and preparation of the final manuscript and revisions. All authors have read and approved the final manuscript.
